# Validation of Vectra 3D Imaging Systems: A Review

**DOI:** 10.3390/ijerph19148820

**Published:** 2022-07-20

**Authors:** Alberto De Stefani, Martina Barone, Sam Hatami Alamdari, Arjola Barjami, Ugo Baciliero, Federico Apolloni, Antonio Gracco, Giovanni Bruno

**Affiliations:** 1 Department of Neuroscience, School of Dentistry, University of Padova, 35100 Padova, Italy; martinabarone4@gmail.com (M.B.); hatamisam97@gmail.com (S.H.A.); arjolabarjami@yahoo.it (A.B.); antonio.gracco@unipd.it (A.G.); giobruno93@gmail.com (G.B.); 2 Maxillofacial Surgery Complex Unit of San Bortolo Hospital of Vicenza, 36100 Vicenza, Italy; baciliero@studiobaciliero.com (U.B.); federico.apolloni@aulss8.veneto.it (F.A.)

**Keywords:** Vectra, Vectra 3D imaging systems, 3D imaging, stereophotogrammetry

## Abstract

**Aim:** Three-dimensional facial imaging systems are a useful tool that is gradually replacing two-dimensional imaging and traditional anthropometry with calipers. In this varied and growing landscape of new devices, Canfield (Canfield Scientific, Parsippany, NJ, USA) has proposed a series of static and portable 3D imaging systems. The aim of this systematic review was to evaluate the current literature regarding the validation of Canfield’s Vectra imaging systems. **Materials and Methods:** A search strategy was developed on electronic databases including PubMed, Web of Science and Scopus by using specific keywords. After the study selection phase, a total of 10 articles were included in the present review. **Results:** A total of 10 articles were finally included in the present review. For six articles, we conducted a validation of the Vectra static devices, focusing especially on the Vectra M5, Vectra M3 and Vectra XT. For four articles, we validated the Vectra H1 portable system. **Conclusions:** All of the reviewed articles concluded that Canfield’s Vectra 3D imaging systems are capable of capturing accurate and reproducible stereophotogrammetric images. Minor errors were reported, particularly in the acquisition of the perioral region, but all the evaluated devices are considered to be valid and accurate tools for clinicians.

## 1. Introduction

Three-dimensional (3D) facial imaging systems are useful tools that enable facial evaluation. As a strong correlation between skeletal and soft-tissue morphology exists, and facial evaluation can be fundamental for pre-surgery planning, postoperative assessment of facial symmetry, and surgical shape changes [1]. Especially in clinical assessments, it enables an accurate diagnosis for different syndromes through the evaluation of normal and abnormal growth, surgical planning and orthodontic treatment [2].

The 3D system has gradually replaced the 2D (two-dimensional) system and traditional anthropometry, which is performed through direct measurements via the use of sliding and spreading calipers. The 2D system has the advantage of being inexpensive, but on the other hand, it requires patients’ collaboration, and it is time consuming [3]. Another important drawback is the fact that it does not allow a record of landmark coordinates or data storage.

Therefore, due to the unavoidable shift toward 3D facial imaging systems and the increasing requirements of imaging for medical treatment, several types of 3D surface acquisition methods have been developed over time. These technologies may be classified into systems related to stereophotogrammetry, laser scanning, structured light, video imaging, radiation sources, MRI and ultrasound [4]. 

Stereophotogrammetry is currently the most promising method of soft-tissue evaluation. It uses high-resolution and fast-acquisition camera systems to capture images of the individual at different angles (the principle of stereoscopy) and reconstructs a 3D image [5]. This system is equipped with inherent software that not only enables the visualization and analysis of the images, but also enables the realization of linear, angular and volumetric morphological measurements. Through the extraction of coordinates x, y and z, it is possible to perform a wide variety of statistical analyses of the shape. Therefore, this acquisition system enables the computation of surface areas, and the registration and superimposition of 3D surfaces. Compared to 2D systems, the 3D facial imaging system has the advantage of allowing indirect anthropometry, thus avoiding physical contact with the individual and, consequently, both reducing the risk of injuries and preventing tissue deformation. It also provides data collection and a permanent record of consultations [5]. Furthermore, photogrammetric devices allow an optimal and accurate representation of skin texture and color [6].

Despite the noticeable and promising advantages of stereophotogrammetry over the traditional anthropometry techniques, the accuracy and reliability of 3D imaging systems has to be established [5].

In the 3D facial imaging systems’ panorama, Canfield (Canfield Scientific, Parsippany, NJ, USA) developed a series of devices called “Vectra”, both in static and portable versions.

As the static devices are expensive, bulky, stationary rigs that require frequent calibration, they allow the acquisition of multiple facial captures simultaneously due to the settings of different cameras at specific angles. Portable devices, which comprise an SLR camera and a laptop computer, are less expensive; however, in order to obtain a final 3D facial model, they require the acquisition of three images of the same subject from different angles and within a limited time period [7]. Since subjects may change facial posture between successive captures or could make involuntary movements, these acquiring systems may present a greater possibility of error in the representation of the final 3D model [8].

Table 1 summarizes the characteristics of Canfield’s Vectra devices described in the literature; most of them are static, except for the Vectra H1, which is the sole portable device.

The aim of this systematic review of the literature was to give an overview on the validity of Canfield’s Vectra devices, both portable and static, in capturing precise and repeatable images of the facial region. To pursue the objective of the study, only articles evaluating facial imaging systems (both stationary and portable) were taken into consideration.

## 2. Materials and Methods

The present systematic review was undertaken according to the PRISMA (Preferred Reporting Items for Systematic Reviews and Meta-Analyses) guidelines [12].

The focused question pursued by researchers to perform this systematic review of the existing literature was “Are VECTRA 3D static and handheld devices able to acquire accurate stereophotogrammetric images of the face?”

### 2.1. Research Strategy

The present systematic review was based on a research strategy that included a detailed examination of articles available on:

- PubMed (www.pubmed.gov);
- Web Of Science (www.webofscience.com) among the following categories: surgery, dermatology, dentistry and oral surgery medicine, medicine research experimental, pathology, and multidisciplinary sciences;
- Scopus (www.scopus.com), among the categories medicine and dentistry.

The research was conducted from August to October 2021. The keywords used were (Vectra) OR (Vectra 3D) OR (vectra system) OR (vectra three-dimensional facial imaging system) OR (vectra three dimensional facial imaging system) OR (vectra 3D facial imaging system) OR (vectra stereophotogrammetry system) OR (vectra scanner) OR (vectra 3D scanning) OR (vectra three-dimensional scanning) OR (vectra camera) OR (vectra 3-dimensional surface imaging) OR (vectra three-dimensional surface imaging).

This research led to 592 articles on Pubmed, 238 articles on Web Of Science and 372 articles on Scopus, amounting to a total of 1202 articles.

### 2.2. Study Selection

All the publications were subjected to selection based on pre-established inclusion and exclusion criteria:

- The first line of exclusion was performed based on the type of article (letters, comments, case reports/series and reviews were excluded); language (only articles in English were included); unavailability of abstract and article; article field (articles not regarding stereophotogrammetry were excluded). This phase led to the exclusion of 897 articles.
- Duplicates were excluded using Clarivate’s EndNote Online; additional duplicates found were removed manually. Articles removed as duplicate totaled 148.
- The second line of exclusion was applied to the remaining studies, which were subjected to abstract analysis; all the articles that were not relevant to the aim of the review were excluded. All the articles that did not validate Vectra devices’ acquisition accuracy in the facial region were excluded. After this process, 144 articles were excluded.
- The remaining 13 articles were subjected to full-text analysis. Three articles were excluded after this analysis: one article since it used software to detect artifacts and biases in the system as opposed as validating its accuracy [13], one as the validation was based on subjective parameters [14], and one because the study was performed on mannequin heads (i.e., not on human subjects) [15].
- Ten articles were included for the final revision. These were thoroughly analyzed by the reviewers and all the relevant data were collected and organized in a table (Microsoft^®^ Office 365^®^ Word).

**Figure 1:**
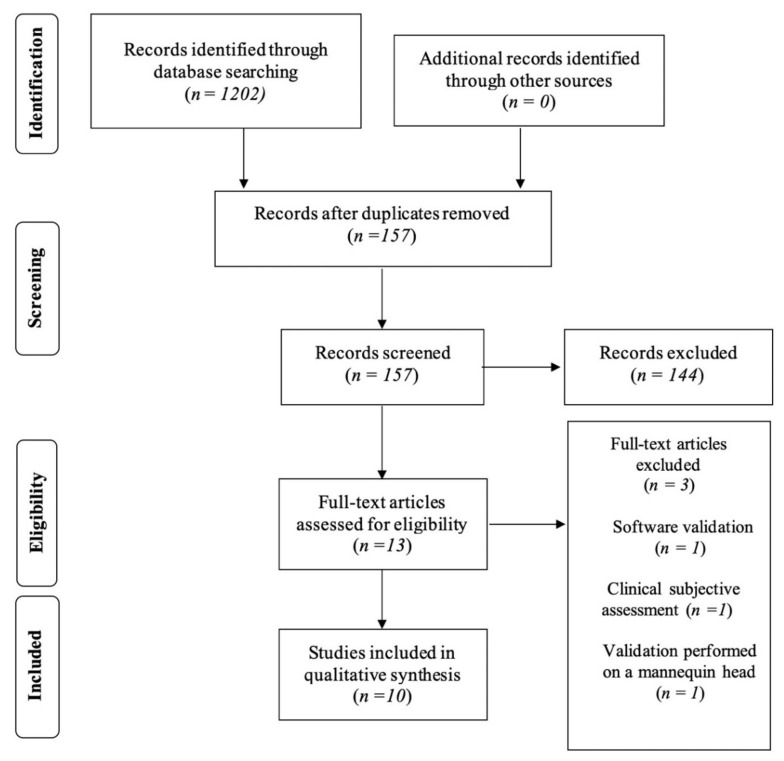
Summary of the phases of study selection showing the number of studies identified, screened, eligible and included in the present review.

Figure 1: Article screening: four-phase PRISMA (*Preferred Reporting Items for Systematic Reviews and Meta-Analyses*) flow diagram for study collection showing the number of studies identified, screened, eligible and included in the present review.

### 2.3. Data Extraction

The methodological characteristics of the selected papers were summarized according to the PICO approach:

- ‘P’ (patients/problem/population)—studied population.
- ‘I’ (intervention)—stereophotogrammetry with Vectra 3D portable or static systems.
- ‘C’ (comparison)—if a comparison with another system was performed.
- ‘O’ (outcome)—evaluation of accuracy and reproducibility of Vectra 3D devices.

### 2.4. Defining Accuracy

According to ISO-5725, accuracy can be divided into trueness and precision, and precision itself can be divided into repeatability and reproducibility.

- Trueness can be defined as closeness of agreement between the expectation of test results and a true value.
- Precision can be defined as the closeness of agreement between independent test results obtained under stipulated conditions and these conditions’ separate repeatability from reproducibility.
  ◦ Repeatability is precision under conditions whereby independent test results are obtained using the same method on identical test or measurement items, in the same test or measuring facility, by the same operator using the same equipment within short intervals of time
  ◦ Reproducibility is precision under conditions whereby independent test results are obtained with the same method on identical test or measurement facilities, with different operators using different equipment.

The following evaluations were conducted using different methods and equipment, and the authors often defined accuracy, trueness, precision, repeatability and reproducibility differently than what is defined by ISO standards.

In this review, ISO definitions were applied to the results, and much of the data acquired from the articles was re-labeled.

## 3. Results

The initial research on the PubMed, Scopus and Web of Science databases led to a total of 1202 potentially relevant articles. The previously stated inclusion criteria were applied in order to select eligible articles to be included in this systematic review. The duplicate removal process led to 157 articles, which were subjected to title and abstract analysis. A total of thirteen articles were included for the full-text analysis; after that, three of them were excluded: the first one because it validated the device through a clinical subjective assessment; the second one as it validated the Vectra integrated software and not the device; and the third one because the study was performed on not human subjects, but on mannequin heads. At the end of the process of selection of the studies, a total of 10 articles were included and reviewed by the authors.

### 3.1. Study Characteristics

The oldest included study was published in 2010 by De Menezes et al. [2], while the most recent was published in 2021 by Liu et al. [9] All the included studies evaluated the precision and reproducibility of Canfield’s Vectra devices. Six articles validated static devices [2,4,5,9,10,11], while four validated the Vectra H1 portable device [1,6,7,8].

### 3.2. Vectra Static Devices 

Among the studies on static devices, two evaluated the Vectra M5 system [4,10], three the Vectra M3 system [2,5,9] and one the Vectra XT system [11]. Table 2 shows the main data extracted from the articles included in this systematic review.

All the studies included a study sample taken from the general population, except that of Othman et al., which evaluated patients with cleft lip and palate [10]. The lowest sample size was that of De Menezes et al. (10 patients) [2], while that of Liu et al. was the highest (40 patients) [9].

Most studies [1,2,4,5,10] used a series of landmarks placed on the face of the subjects and calculated the linear distances between these points, while Andrade et al. [5] considered angular measurements, too. Liu et al. [9] placed a series of small objects of different areas in the orbital region, while Verhulst et al. [11] matched the surface of the 3D images and used color-coded heat maps to determine the variation.

In order to evaluate trueness, the results obtained by the device were compared with those obtained by a caliper [2,9,10] or by other 3D imaging devices [11], while three studies [3,4,5] did not make a comparison with another system. Only Verhulst et al. [11] compared the Vectra device analyzed (i.e., Vectra XT) with other two imaging devices, specifically, with 3dMDface (3dMD, Atlanta, GA) and Artec Eva (Artec, Luxembourg). They asserted that the differences in the reconstruction of the surfaces made by the three imaging systems were significant, but without clinical relevance (as all <0.5 mm), so the accuracy of the 3D images obtainable by the three systems was considered comparable.

Although some measurements have lower rates of repeatability and have slightly higher deviations from those obtained via caliper (particularly around the mouth [5,10]), all the reviewed studies suggest that Vectra devices represent reliable 3D imaging systems. All the authors of the included studies agreed that Vectra static devices are precise and reproducible systems.

### 3.3. Vectra H1

Four of the eleven articles reviewed evaluate Canfield’s portable Vectra H1 imaging device [1,6,7,8]. The collected results are shown in Table 3.

All the studies evaluated a study sample taken from the general population. Liberton et al. [1] considered children, too. The sample size of Savoldelli et al. [6] was the lowest (only 2 patients), while that of Gibelli et al. [8] was the highest (50 patients).

All the included studies used a series of measurements obtained by calculating the distance between a set of landmarks placed on the faces of the patients. However, Gibelli et al. [8] considered angular, facial surface and facial volume measurements, too, while Camison et al. [7] also used heat maps.

The considered measurements were compared with those obtained using a caliper [6] or another static 3D imaging device [1,7,8]. In particular, Liberton et al. compared the Vectra H1 device with the 3dMD face system and the ProFace laser scanning system, while Gibelli et al. [8] compared it with the Vectra M3 (i.e., a static 3D imaging system from the same producer) and Camison et al. [7] with the 3dMD face system.

All studies deemed the device accurate and comparable to fixed devices, and the obtained results are considered acceptable for clinical practice, yet a small degree of errors is present.

## 4. Discussion

3D stereophotogrammetry is quickly acquiring popularity thanks to its acquisition speed and the possibility of taking measurements on the acquired images, rather than the patient. These images and the data that they provide can be used in various scenarios that range from evaluating the progression and follow-up of various therapies [16,17] to predicting the outcome of surgical treatments such as rhinoplasty [18]. Within this growing market for devices, Canfield offers a set of 3D imaging systems—the Vectra devices—both in stationary and portable form. Yet, in order to be considered valid and reliable tools for clinicians, a thorough evaluation of their accuracy is required.

The aim of the present systematic review of the literature was to assess the validity of Canfield’s Vectra static and portable devices in acquiring accurate 3D volumetric images of the face.

Most of the included studies [2,4,5,6,7,8,9,10,11] evaluated the devices on adult patients, while Liberton et al. [1] evaluated the Vectra H1 on 10 subjects ranging from 8 to 30 years old; thus, in their study sample, they included some children, too. Only one study was not performed on the general non-syndromic population; Othman et al. [10], in fact, analyzed Canfield’s Vectra M5 devices on patients with cleft lip and palate.

Patient compliance was not addressed in these studies, probably because, in almost all of them, the study sample included an adult population. Moreover, the performance of this device on pediatric patients still needs to be completely assessed. More studies on pediatric and non-compliant subjects would be useful.

3D stereophotogrammetry is an innovative tool that allows clinicians to quickly collect and thoroughly examine volumetric images of facial soft tissues. Most of the studies included in the present systematic review compared linear or angular measurements between landmarks [1,2,4,5,6,7,8,10], while Verhulst et al. [11] used the surface-matching of different 3D images of the same patient to evaluate intersystem reproducibility and intrasystem repeatability, and Liu J et al. [9] placed small objects (of known dimensions) on the periocular region. Gibelli et al. [8] used both linear and angular measurements between landmarks, as well as surface and volume measurements of matched surfaces.

The authors of the included studies determined the trueness of the devices’ measurements by placing landmarks or small objects on the subjects’ faces and comparing the linear distances and angles between them (or the size of the objects) with the results obtained from physical measurements using a caliper.

The repeatability was evaluated by comparing the results obtained from multiple captures. In particular, Verhulst et al. [11] and Camison et al. [7] assessed the accuracy of the systems by matching the 3D images and generating heat maps that highlighted the areas with the highest and lowest discrepancies.

Canfield’s Vectra 3D imaging devices were evaluated positively in all the reviewed studies, both in the static and portable form. The facial region where the least true and repeatable measurements seemed to be recorded is the mouth area [5,10]. This evidence could be caused by slight alterations of the position of the lips caused by mimic muscles during different recordings or when compared to the caliper. It must be mentioned that the study of Othman et al. [10] was conducted on patients with cleft lip and palate, where the presence and severity of the condition can affect the accuracy of the measurements that involve the upper lip. This was asserted by the authors of the study themselves. Verhulst et al. [11] also reported the region of the eyes to have significant errors in Vectra 3D imaging, and for this reason, they excluded it from their analysis. In any case, these devices can be considered to be valid tools for clinical practice.

Both the static and portable devices proved to be capable of acquiring precise 3D images of the face, but potential differences between the systems were not assessed. The Vectra H1 portable device is less expensive than the static ones, but it requires three acquisitions, meaning more time spent using it, if compared to the single acquisition needed by the static devices [7]. This could negatively affect the precision of the captured 3D image. Nevertheless, the Vectra H1 proved to be accurate in all the reviewed studies. Furthermore, something that can be considered is that a portable device can be useful and convenient in certain situations and contexts—for example, because, unlike a static device, it can be moved between different departments or structures, both for clinical and research reasons.

Although to evaluate the precision and reproducibility of a device these types of analyses are necessary, it should be noted that these devices and their associated software are capable of significantly more complex evaluations of the face. Once their reliability is assessed, Vectra 3D imaging systems could be used to their full potential, taking advantage of the various tools they offer, such as surface matching, volumetric evaluations and comparisons of 3D images and surgical simulations.

These systems could prove useful in different medical branches, ranging from oral and maxillary surgery to plastic surgery, orthodontics, prosthodontics and, ideally, any field of study that implies the evaluation of facial soft tissues. They can implement the digital workflow of clinicians. Their use for surgical planning has been described for procedures such as rhinoplasty [18] and blepharoplasty [19]. Thanks to their integrated software, Vectra devices could allow soft-tissue-based treatment planning.

## 5. Strengths and Limitations of the Study

This study evaluates and reviews a wide array of validation articles. The authors of the reviewed studies analyzed different Vectra 3D devices and used various methods to evaluate the reproducibility and accuracy of these systems. The devices went through various tests and consistently yielded positive results. Nevertheless, this variety of evaluations comes at a cost: the difficulty in standardizing many of the results. Given the heterogeneity of methods, device and statistical analyses, a direct comparison of many of the results yielded by the included studies could not be performed.

It is also worth mentioning that the Vectra devices are constantly being updated and new models are being released. For example, at the time of this review, the Vectra H1 imaging system is no longer available and has been replaced with the more recent Vectra H2. However, no validation article of this system was found in the current literature.

The present review has methodological limitations, due to the “narrative” purpose: the absence of scientific evidence on the accuracy of VECTRA leads to the departure from a rigorous, systematic approach. This study seeks to satisfy the need for “ground knowledge” on this topic and offer practical hints for developing further investigations. Furthermore, PROSPERO registration was absent for this paper.

## 6. Conclusions

Canfield’s Vectra imaging systems (i.e., Vectra M5, Vectra M3, Vectra XT and Vectra H1) are devices capable of acquiring precise and reproducible 3D volumetric images of the face, with slight imprecision in the perioral region. More studies assessing the validity of these systems on pediatric or syndromic subjects or those with cranio-facial anomalies, and on non-compliant subjects, would be needed.

## Figures and Tables

**Table 1:** Canfield’s Vectra devices.

Vectra Device	Calibration	Features
**H1**	Does not require a pre-calibrated coordinate system. Features a targeting system consisting of two converging green lights projected onto the subject’s face. Overlap of both lights indicates the correct shooting distance was obtained.	A single camera. A non-ionizing and handheld device that does not require any specific environment. It requires three consecutive acquisitions (two three-quarter profiles and a frontal one) in order to generate a 3D model [1].
**M3**	Needed	Six cameras divided into three modules assembled on a triangular rigid structure. The capture system has a geometric resolution of 1.2 mm (polygon edge length), 3.5 milliseconds of capture time, intelligent flash units (on-board modular), passive stereophotogrammetry technology, ground support, a footstool and a Dell computer [9].
**M5 360**	Needed	Five pods of cameras placed at different angulations from the subject. Each pod contains one color camera and one monochrome camera. These two-dimensional (2D) digital cameras capture the image simultaneously. Capture time is less than 2 ms [10].
**XT**	Needed	Three pods with a total of six cameras [11].

**Table 2:** Articles validating Canfield’s Vectra static devices. (Original definitions are maintained. Results are re-labeled in italic with definitions according to ISO-5725).

**N°**	**Author/Year**	**Patients/Problem/Population**	**Intervention**
1	Liu J. et al., 2021 [9]	40 subjects (20 Caucasian and 20 Chinese)	- System used: Vectra M3 imaging system (images of the periocular region). - Four objects of different dimension were placed in the middle of the lower eyelid. - Object 5 consisted of seven smaller objects placed in the periorbital region around the eye. - Paired *t*-tests and Wilcoxon tests were used to analyze differences between direct and 3D values (*trueness*).
2	Othman SA. et al., 2019 [10]	37 cleft patients, 20 M, 17F, m.a. 23, 84 years.	- System used: Vectra M5 imaging system. - A total of 16 landmarks were positioned on the patient. - A total of 19 linear distances between landmarks were calculated. - Results obtained by the system were compared with those obtained by a caliper (trueness). - Paired *t*-tests, Wilcoxon and ICC tests were performed to assess whether repeated measurements were significantly different at specific anatomic landmarks (repeatability). - To evaluate the reliability of the measurements per observer and between two observers, the same tests were assessed (reproducibility).
3	Verhulst A. et al., 2017 [11]	15 subjects, 6M and 9F, m.a. 37 ± 12 years.	- Systems used: 3dMDface, Vectra XT, Artec Eva. - Three images were captured for each 3D Imaging system. No landmarks, surface matching. - To determine the reproducibility of every system (intrasystem accuracy), the first 3D image of every patient was matched onto the second 3D image for every imaging system, separately. This was performed using a surface-based matching algorithm. - To determine the differences between the two 3D images, from each point on the first image, the closest distance from the second image was calculated. The differences between the images were visualized by making a color-coded heat map (repeatability). - To determine the differences between the imaging systems (addressed as intersystem accuracy), the 3D images obtained using Vectra XT and Artec Eva were matched onto the 3D images obtained by the 3dMDface system. This does not count as trueness as none of the systems produced values defined by the authors as a true value. It was not considered reproducible as the imaging system cannot be considered a variable in the system validation itself.
4	Andrade LM. et al., 2017 [5]	30 Brazilian adults, 5M and 25F, m.a. 26.7 ± 5.63.	- System used: Vectra M3. Two Images of each participant were captured with an interval of 1 week. - Eleven landmarks were placed on the participants’ faces. - Nine angular and two linear measurements were taken. - Repeatability: multiple captures from the same device were analyzed using the mean absolute differences (MAD), REM, TEM, ICC and Bland-Altman analysis. Paired *t*-tests sought any systematic errors between the acquisitions.
5	Othman SA. et al., 2013 [4]	30 Semai adults, 15M and 15F, aged 20 to 25 years	- System used: Vectra M5 imaging system. - A total of 24 landmarks were manually placed by an operator on the captured images. Distance between landmarks was calculated. - The ICC test was used to determine the intra-examiner reproducibility of the two readings (repeatability). A paired *t*-test or a Wilcoxon Rank test was performed for each landmark, based on the normality of the distribution.
6	De Menezes M. et al., 2010 [2]	10 healthy adults, 5M and 5F, aged from 20 to 30 years.	- System used: Vectra M3 imaging system. - A total of 50 soft-tissue landmarks were placed on the patients’ faces and the distances between the landmarks were calculated. - To analyze accuracy, measurements of a 6 cm cubic box were made (trueness). - To analyze the inter-operator error, the same landmarks were placed and referenced by two separate operators (reproducibility). - To analyze the reproducibility after subject repositioning, the subjects were included in two acquisitions.
**N°**	**Comparison**	**Outcome**	
1	Results were compared with a caliper	- The mean area deviation of all objects was less than 0.02 cm^2^. - Caucasian patients had a lower accuracy than Chinese patients, and M had a lower accuracy than F. - *t*-test and Wilcoxon test showed no statistically significant difference between the two measurement methods, except for objects 1 and 5.	
2	Results were compared with a caliper	- No statistically significant difference between direct anthropometric measurements and results from Vectra M5 360 was shown in most measurements. - Measurement of nose width and upper vermilion height showed a deviation higher than ±2 mm. - Paired *t*-tests showed that none of the linear measurements between the two observers were statistically significant. - ICC test showed that nine measurements had good agreement between observers, seven were in the range of acceptable and three had poor agreement.	
3	Results were compared with 3dMDface system and Artec Eva	- The highest reproducibility (repeatability) was shown by 3dMDface system (0.18 ± 0.15 mm) and Vectra XT (0.15 ± 0.15 mm), with no significant difference between each other. The Artec Eva reproducibility (repeatability) was 0.26 ± 0.24 mm, which was significantly different from the other two systems. - The Vectra XT showed a mean difference with the 3dMDface system of 0.32 ± 0.26, which was significantly different. The Artec Eva showed a mean difference with the 3dMDface system of 0.44 ± 1.09 mm, which was significantly different. - The differences found between systems were significant, but not clinically relevant, as they were all <0.5 mm.	
4	None	- MAD: three measures exceeded the clinical limit of 2.0 mm or 2°. The nasolabial angle (2.438), mentolabial angle (3.348) and the linear measurement LFH (2.13 mm). - REM: one moderate measure (9.1%), three good measures (27.3%), five very good measures (45.5%) and two excellent measures (18.2%). - TEM: the nasolabial (2.178) and mentolabial (2.888) angles showed values > 2.08. - ICC: the only result that was in the rating limit was the mandibular angle, with an outcome considered poor. - The paired *t*-test showed a significant difference in the MFH, indicating a systematic error in this variable.	
5	None	- ICC for all 24 landmarks ranged from 0.68 to 0.97 and indicated moderate-to-high reliability and reproducibility (repeatability) of all soft-tissue landmarks. - Paired *t*-tests and Wilcoxon Rank tests revealed that there were no significant differences in the identification of all 24 landmarks.	
6	None	- All measurements of the box were very accurate in regard to the values obtained with a caliper (linear SD 0.03, angular SD 0.19 and area SD 0.01). - No systematic errors between measurements obtained using the two different calibrations were found. - Data obtained via two different operators had negligible random errors, with MAD values ranging from 0.05 mm and 0.9 mm, and all TEM values were lower than 0.7 mm. - When the same subjects were measured twice with the same calibration, the MADs were typically less than 1.0 mm, except for mouth width. TEM also scored higher when measuring mouth width.	

**F**—female/s, **M**—male/s, **m.a.**—mean age, **ICC**— intra-class correlation coefficient, **MAD**—mean absolute differences, **TEM**—technical error of magnitude, **REM**—relative error of measurement.

**Table 3:** Articles validating Canfield’s Vectra H1 portable device. (Original definitions are maintained. Results are re-labeled in italic with definitions according to ISO-5725).

**N°**	**Author/Year**	**Patients/Problem/Population**	**Intervention**
1	Savoldelli C. et al., 2019 [6]	2 adults, 1M and 1F, m.a. 23 years.	- A total of 11 landmarks were placed on the patients’ faces using a dermographic pencil. - A total of 23 distances among the landmarks were defined and measured. - Accuracy (trueness), repeatability and reproducibility were calculated.
2	Liberton DK. et al., 2019 [1]	10 subjects (adults and children), 3M and 7F, m.a. 30 years.	- A total of 21 landmarks were placed on each image. - Three landmarking trials were performed on each patient for each imaging system, for a total of 90 landmark sets. - Two three-quarter profile images were acquired with the 3dMD face system and aligned into a single facial surface. - Two three-quarter profiles and a frontal one were sequentially acquired using the Vectra H1 system. - The ProFace surface images were acquired using a cone-beam computed-tomography machine. - Results from Vectra H1 and ProFace were compared to those obtained from 3dMD face system, which were considered true values (trueness)
3	Gibelli D. et al., 2018 [8]	50 adults, 16M and 34F, aged between 19 and 61 years.	- A total of 50 landmarks were marked on each participant’s face using liquid eyeliner. - Every subject underwent four facial scans (two for each acquisition system). - A total of 15 linear distances, 12 angles, facial surface and facial volume measurements were verified both within the device and between devices using Bland-Altman test and calculation of TEM and REM. - The two scans obtained using the same device were registered and superimposed to calculate RMS (point-to-point) distance between the two surfaces (repeatability) - Scans obtained using different devices were matched to calculate RMS (point-to-point) distance between the two surfaces. (As both systems are Vectra devices, this can be considered intersystem reproducibility.)
4	Camison L. et al., 2017 [7]	26 adults, 6M and 20F, m.a. 33.1 years.	- A total of 17 landmarks were placed on the patients’ faces. - A total of 136 linear distances between landmarks were calculated. - Heat maps were generated for each 3D facial surface previously registered. - Results were compared with those yielded by the 3dMD face system, which were considered true (trueness)
**N°**	**Comparison**	**Outcome**	
1	Results were compared with a caliper	- Repeatability (measured as intra-operator variation): the systematic error (and standard deviation) was 0.96 mm (0.89). - Reproducibility (measured as inter-operator variation): the systematic error (and standard deviation) was 0.53 mm (0.43). - Accuracy (trueness): Vectra H1 was highly accurate as it showed negligible systematic error. - These results are fully acceptable for clinical practice.	
2	Results were compared with results from the 3dMD face system and with the ProFace laser scanning system.	- ANOVA found that error due to landmarking trial was not significant and the proportion of the overall shape variance explained by digitation was only 0.88%. - The ANOVA term for error due to imaging system was also not significant and the proportion of variance explained by the imaging system was 3.11%. - For 16 out of 21 landmarks, the 3dMD and Vectra systems had the smallest mean differences. - The 3dMD, Vectra H1 and ProFace scanners are comparable were validated for basic and clinical research, though ProFace had slightly more variability since it took longer to complete the image acquisition.	
3	Results were compared with results from Vectra M3 (static)	- Most linear, angular and surface area measurements had a high repeatability in M3-M3, H1-H1 and M3-H1 comparisons, ranging between 82.2% and 98.7% (TEM range: 0.3–2.0 mm, 0.4–1.8 deg; REM range: 0.2–3.1%). - Volumes and RMS distances showed evident differences passing from M3-M3 (on average, 0.22 mm; SD: 0.14) to H1-H1 (on average, 0.44 mm; SD: 0.36) comparisons, and reached the maximum when scans from the two different devices were compared. - Vectra H1 proved to be reliable for assessing linear measurements, angles and surface areas. On the other hand, the influence of involuntary facial movements on volumes and RMS distances are more important in comparison with Vectra M3.	
4	Results were compared with results from the 3dMD face system. Comparison was performed on both live patients and a static mannequin head.	- The 136 distances were highly comparable between the two cameras. - The differences were within a ±1 mm threshold. The average TEM value was 0.84 mm. The average RMS value of the 26 surface-to-surface comparisons was 0.43 mm. - The results indicate that the Vectra H1 system is sufficiently accurate for most clinical applications, since errors smaller than 2 mm are generally considered appropriate for what concerns accuracy and precision in 3D photogrammetric validation.	

**F**—female(s), **M**—male(s), **m.a.**—mean age, **TEM**—technical error of magnitude, **REM**—relative error of measurement, **RMS**—root mean square.

## Data Availability

Not applicable.

## References

[1] Liberton D.K., Mishra R., Beach M., Raznahan A., Gahl W.A., Manoli I., Lee J.S. (2019). Comparison of Three-Dimensional Surface Imaging Systems Using Landmark Analysis. J. Craniofacial Surg..

[2] de Menezes M., Rosati R., Ferrario V.F., Sforza C. (2010). Accuracy and Reproducibility of a 3-Dimensional Stereophotogrammetric Imaging System. J. Oral Maxillofac. Surg..

[3] Gibelli D., Pucciarelli V., Poppa P., Cummaudo M., Dolci C., Cattaneo C., Sforza C. (2018). Three-dimensional facial anatomy evaluation: Reliability of laser scanner consecutive scans procedure in comparison with stereophotogrammetry. J. Cranio-Maxillofac. Surg..

[4] Othman S.A., Ahmad R., Mericant A.F., Jamaludin M. (2013). Reproducibility of facial soft tissue landmarks on facial images captured on a 3D camera. Aust. Orthod. J..

[5] Andrade L.M., Rodrigues da Silva A.M.B., Magri L., Rodrigues da Silva M.A.M. (2017). Repeatability Study of Angular and Linear Measurements on Facial Morphology Analysis by Means of Stereophotogrammetry. J. Craniofacial Surg..

[6] Savoldelli C., Benat G., Castillo L., Chamorey E., Lutz J.-C. (2019). Accuracy, repeatability and reproducibility of a handheld three-dimensional facial imaging device: The Vectra H1. J. Stomatol. Oral Maxillofac. Surg..

[7] Camison L., Bykowski M., Lee W.W., Carlson J.C., Roosenboom J., Goldstein J.A., Losee J.E., Weinberg S.M. (2018). Validation of the Vectra H1 portable three-dimensional photogrammetry system for facial imaging. Int. J. Oral Maxillofac. Surg..

[8] Gibelli D., Pucciarelli V., Cappella A., Dolci C., Sforza C. (2018). Are Portable Stereophotogrammetric Devices Reliable in Facial Imaging? A Validation Study of VECTRA H1 Device. J. Oral Maxillofac. Surg..

[9] Liu J., Guo Y., Arakelyan M., Rokohl A.C., Heindl L.M. (2020). Accuracy of Areal Measurement in the Periocular Region Using Stereophotogrammetry. J. Oral Maxillofac. Surg..

[10] Othman S.A., Saffai L., Hassan W.N.W. (2020). Validity and reproducibility of the 3D VECTRA photogrammetric surface imaging system for the maxillofacial anthropometric measurement on cleft patients. Clin. Oral Investig..

[11] Verhulst M.A., Hol M., Vreeken B.R., Becking A., Ulrich D., Maal T. (2018). Three-Dimensional Imaging of the Face: A Comparison Between Three Different Imaging Modalities. Aesthetic Surg. J..

[12] Moher D., Liberati A., Tetzlaff J., Altman D.G. (2010). Preferred reporting items for systematic reviews and meta-analyses: The PRISMA statement. Int. J. Surg..

[13] White J.D., Ortega-Castrillon A., Virgo C., Indencleef K., Hoskens H., Shriver M., Claes P. (2020). Sources of variation in the 3dMDface and Vectra H1 3D facial imaging systems. Sci. Rep..

[14] Ueda N., Imai Y., Yamakawa N., Yagyuu T., Tamaki S., Nakashima C., Nakagawa M., Kirita T. (2021). Assessment of facial symmetry by three-dimensional stereophotogrammetry after mandibular reconstruction: A comparison with subjective assessment. J. Stomatol. Oral Maxillofac. Surg..

[15] Metzler P., Sun Y., Zemann W., Bartella A., Lehner M., Obwegeser J.A., Kruse-Gujer A.L., Lübbers H.-T. (2013). Validity of the 3D VECTRA photogrammetric surface imaging system for cranio-maxillofacial anthropometric measurements. Oral Maxillofac. Surg..

[16] Michaux D., Van de Casteele E., Dielen D., Van Hemelen G., Nadjmi N. (2021). The effect of subspinal Le Fort 1 corticotomy on nasal morphology in surgically assisted rapid palatal expansion. Int. J. Oral Maxillofac. Surg..

[17] van der Vlis M., Dentino K.M., Vervloet B., Padwa B.L. (2014). Postoperative Swelling After Orthognathic Surgery: A Prospective Volumetric Analysis. J. Oral Maxillofac. Surg..

[18] Persing S., Timberlake A., Madari S., Steinbacher D. (2018). Three-Dimensional Imaging in Rhinoplasty: A Comparison of the Simulated versus Actual Result. Aesthetic Plast. Surg..

[19] Miranda R.E., Matayoshi S. (2021). Vectra 3D Simulation in Lower Eyelid Blepharoplasty: How Accurate is it?. Aesthetic Plast. Surg..

